# Evaluating the Consistency of Current Mainstream Wearable Devices in Health Monitoring: A Comparison Under Free-Living Conditions

**DOI:** 10.2196/jmir.6874

**Published:** 2017-03-07

**Authors:** Dong Wen, Xingting Zhang, Xingyu Liu, Jianbo Lei

**Affiliations:** ^1^ Peking University Third Hospital Beijing China; ^2^ Center for Medical Informatics Peking University Beijing China; ^3^ Department of Prosthodontics Affiliated Hospital of Stomatology Southwest Medical University Luzhou China; ^4^ School of Medical Informatics and Engineering Southwest Medical University Luzhou China

**Keywords:** fitness trackers, monitoring, physiologic, motor activity, activities of daily living, health status

## Abstract

**Background:**

Wearable devices are gaining increasing market attention; however, the monitoring accuracy and consistency of the devices remains unknown.

**Objective:**

The purpose of this study was to assess the consistency of the monitoring measurements of the latest wearable devices in the state of normal activities to provide advice to the industry and support to consumers in making purchasing choices.

**Methods:**

Ten pieces of representative wearable devices (2 smart watches, 4 smart bracelets of Chinese brands or foreign brands, and 4 mobile phone apps) were selected, and 5 subjects were employed to simultaneously use all the devices and the apps. From these devices, intact health monitoring data were acquired for 5 consecutive days and analyzed on the degree of differences and the relationships of the monitoring measurements ​​by the different devices.

**Results:**

The daily measurements by the different devices fluctuated greatly, and the coefficient of variation (CV) fluctuated in the range of 2-38% for the number of steps, 5-30% for distance, 19-112% for activity duration, .1-17% for total energy expenditure (EE), 22-100% for activity EE, 2-44% for sleep duration, and 35-117% for deep sleep duration. After integrating the measurement data of 25 days among the devices, the measurements of the number of steps (intraclass correlation coefficient, ICC=.89) and distance (ICC=.84) displayed excellent consistencies, followed by those of activity duration (ICC=.59) and the total EE (ICC=.59) and activity EE (ICC=.57). However, the measurements for sleep duration (ICC=.30) and deep sleep duration (ICC=.27) were poor. For most devices, there was a strong correlation between the number of steps and distance measurements (R^2^>.95), and for some devices, there was a strong correlation between activity duration measurements and EE measurements (R^2^>.7). A strong correlation was observed in the measurements of steps, distance and EE from smart watches and mobile phones of the same brand, Apple or Samsung (*r*>.88).

**Conclusions:**

Although wearable devices are developing rapidly, the current mainstream devices are only reliable in measuring the number of steps and distance, which can be used as health assessment indicators. However, the measurement consistencies of activity duration, EE, sleep quality, and so on, are still inadequate, which require further investigation and improved algorithms.

## Introduction

### Importance of Monitoring Physical Activities

The amount and patterns of physical activity are closely related to health status and the rehabilitation of chronic conditions. Pedometers have a significant effect on promoting physical exercise. Being sedentary is a significant risk factor for chronic conditions; independent of physical activity and other potential confounders, it is significantly associated with the development of diabetes and other chronic conditions [1]. The longer the daily sedentary time is, the higher the risk of all-cause mortality [2]. The level of activity can be determined by the number of steps per day—5000 steps per day suggest a sedentary state, whereas 10,000 steps per day suggest an active state [3]. For healthy people, increasing the number of steps reduces the risk of death [4]. For patients with chronic conditions, increased activity shows a significant rehabilitation value; for example, middle-aged diabetic patients significantly improved their insulin sensitivity by increasing the number of steps per day during a 5-year follow-up period [5]. However, studies found that elderly populations with a high incidence of chronic conditions often overestimated their physical activity compared with the actual measurement [6]; in this regard, a pedometer can accurately estimate the amount of activity to prompt the user to engage in more physical activities and significantly lower their body mass index (BMI) and blood pressure [7]. The physical activities promoted by a pedometer have been mainly based on the social cognitive theory, self-efficacy theory, and cross-theoretical model, and it was revealed that the outcome of increased physical activity has been achieved by adopting strategies such as setting goals and participating in group activities, and so on [8].

### Importance of Wearable Devices

Wearable devices have richer functions than traditional pedometers and can monitor more health indicators than pedometers do and are gradually replacing traditional pedometers. Supported by a variety of sensors and the increasing computing power, they also have other health monitoring functions [9,10]. Wearable devices for everyday health monitoring are also called “fitness trackers”. Currently, sensors in fitness trackers include three-axis accelerometers, three-axis gyroscopes, three-axis magnetic sensors, optical heart rate sensors, altimeters, ambient light sensors, temperature sensors, bioelectrical impedance sensors, and capacitive sensors. The basic mechanism of the step-counting function is that the acceleration values ​​on 3 orthogonal axes are acquired, from which the secondary wave peaks are monitored after the waves are filtered, and the number of peaks is the number of steps [11]. Compared with traditional physical measurement measures, wearable devices reduce wear discomfort, provide appropriate interactions to facilitate habit development, and capture users’ continuous movement and health data so that the fluctuating trends of users’ health characteristics are better portrayed and potential health risks are spotted in a timely manner [12,13].

### Performance of Current Wearable Devices

Wearable devices are gaining increasing market attention, but the accuracy and reliability of the monitoring of the devices remains inadequate. On one hand, the number of steps, distance, and energy expenditure (EE) have been accurately calculated in a laboratory setting. For example, Takacs et al [14] evaluated the accuracies of the number of steps and speed measurements of 30 subjects wearing multiple Fitbit Ones (Fitbit Inc) under different intensities of physical activity and found that the measurement of the number of steps was reliable and valid (ICC>.95), with an error rate of less than 1.3%; the measurement of distance was reliable but sometimes invalid, particularly at low speeds (*r*>.90), with a rather high error rate of up to 39.6%. LEE et al [15] investigated the accuracy of EE measurements on 60 subjects wearing 8 types of wearable devices performing activities at different intensities and showed that the mean absolute percent error of different devices on EE measurements varied between 9.3% and 23.5%; moreover, the error rate of the Fitbit product was approximately 10% and had a high correlation with the standard value (*r*=.81); the measurements were mostly accurate. On the other hand, in the state of normal life activities, Ferguson et al [16] assessed the reliabilities of the number of steps, activity duration, sleep duration, and EE in 21 subjects continuously wearing 7 types of devices for 48 h and found that for the number of steps and sleep duration, the measurements by the consumer products, and the professional equipment exhibited strong correlations (*r*>.8), but only fair correlations were obtained regarding activity duration and EE (*r*=.52-.91). Moreover, the measurement errors of the number of steps and sleep duration were rather low (<10%), and the measurement error of EE was fair (<30%), whereas that of activity duration was rather high (26-298%). Rosenberger et al [17] investigated the error rates of the measurements of 40 subjects wearing 9 types of devices on sedentary behavior, varying degrees of physical activity, the number of steps, and total sleep duration. They showed that all the devices exhibited a rather high error on each indicator, and no device was able to accurately acquire the activity data for 24 consecutive hours; moreover, the measurement for sleep displayed the smallest error at 8.1%, and that for moderate to vigorous physical activity (MVPA) displayed the largest error at 92%. These results suggest that various monitoring measurements presented by various wearable devices should be considered with caution.

### Significance of This Study

With the rapid development of new products and new functions, the consistencies of health monitoring measurements of different wearable devices in the market must continuously be verified to reach a more uniform assessment. The aforementioned studies show that the reliability of wearable devices and the accuracy and reliability of the measurements of each indicator are still problematic, and such studies are still in the early stages; moreover, emerging products and the validation of measurements in various physical activity states must be considered. In this study, newly launched and representative smart watches, internationally renowned smart bracelets, and popular smart bracelets in China were included, and for the first time, the measurements by mobile phone apps were compared with those obtained from professional equipment. Each subject continuously wore all the devices as they participated in normal life activities, and the 5-day monitoring data were acquired. The aim of the study was to evaluate the degrees of consistency of health monitoring measurements of the mainstream wearable devices in the market and analyzing the discrete degrees and correlations of multiple monitoring measurements of the daily monitoring data under the state of normal life activities, to provide implications to the industry in overcoming difficulties in product development, and to support consumers in choosing the right product.

## Methods

### Selection of Devices and Indicators

Overall, 6 mainstream wearable devices and 4 major apps were chosen for this study. Representative health tracking devices were selected from 3 product categories: smart watches, smart bracelets, and mobile phone apps. First, when choosing smart watches, given that the functions of smart watches and mobile phones were close and that mobile phones from Samsung and Apple were leading products in the market, Samsung Gear S (Samsung Inc) and Apple Watch (Apple Inc) were chosen to represent the smart watches. Second, when choosing the internationally renowned smart bracelets, according to market research data by NPD and Canalys [18-20], Fitbit had the largest market share, and Jawbone also exhibited a good market performance; therefore, Fitbit Surge (Fitbit Inc) and Jawbone Up3 (Jawbone Inc) were chosen to represent the foreign-made smart bracelets. Third, when choosing the Chinese brand smart bracelets, the shipment of the Mi Band in the health tracking devices market has been second to that of Fitbit; furthermore, according to the ranking of smart bracelet products on Zhongguancun online, one of the China’s IT professional websites, Huawei was on top. Thus, the Mi Band (Mi, China) and TalkBand B2 (HUAWEI, China) were chosen to represent the Chinese brand bracelets. Regarding mobile phone apps, the ranking of health and fitness apps was used as the reference, and Pacer and Ledongli were chosen as representatives. Fourth, the mobile phones used in this study were the Samsung Note 3 and the iPhone 6 Plus. To compare the branded smart watches and the preinstalled mobile phone apps, Samsung S Health apps and Apple Health apps were also included.

With respect to indicators, the measurement indicators shared by all the devices, that is, number of steps, distance, activity duration, EE, sleep duration, and deep sleep duration were chosen as the assessment criteria in this study after listing and comparing the available monitoring indicators of all the devices.

### Experimental Procedures

To ensure compliance of the study, 5 subjects from the close community of the research team were recruited via convenience sampling, and the inclusion criteria were as follows: older than 18 years of age and without major critical illnesses, not allergic to rubber straps, able to continuously wear the devices, and willing to participate in the investigation. The subjects were required to wear all devices simultaneously while maintaining normal living conditions for 5 consecutive days including weekends. We hope that the type of activities were able to represent the normal living conditions of the majority of working people, so the main types of activities were supposed to include walking, climbing stairs, sedentary, and so on, as well as light to moderate exercise. In addition, the study duration required inclusion of weekends, to ensure that the forms, amounts and intensity of activities varied from weekdays and can basically represent a complete cycle of the normal activities of the normal people in a real-life environment. As wearing 6 devices simultaneously had a certain challenge, wearing precautions and error prevention measures were instructed to subjects in details. Bracelets and watches were worn on the wrist; the Mi Band bracelet and apple watch were needed to be set correctly for left-right hand mode to ensure the position and settings to maintain consistency. Samsung watch and Fitbit Surge were needed to be manually set to enter or exit the sleep mode; the phone was placed in the pocket of clothes. In addition to the Mi Band bracelet which was said to have battery power for 20 days, other devices are unified charged every night. Data were synchronized between wearable devices and mobile app once a day and the intact data were acquired from the subjects’ devices for each of the 5 consecutive days. This study was approved by the Biomedical Ethics Committee of Peking University, and the subjects were aware of the purpose and process of the study.

### Data Management and Analysis

During the data collection period, the subject manually opened the corresponding app to sync the data. The monitoring data recorded by each of the apps were transcribed to an Excel (Microsoft) spreadsheet at the end point of each subject’s data collection and double-checked. First, a box plot was generated for each measurement indicator to observe the data distribution, and the quartiles of the upper and lower margins, upper and lower quartiles, and median were generated for the measurements ​​of each wearable device on each indicator based on the quartile, which made it possible to visually observe the entire picture for multiple sets of data and to compare the distribution pattern of the measurements. To determine the consistency of the different wearable devices, the intraclass correlation coefficient (ICC) was calculated for each wearable device for each indicator and was used to evaluate interobserver reliability, which ranged from 0 to 1. Values lower than .4 represented a poor reliability, and values higher than .75 represented a good reliability. To observe the discrete degree of the daily measurements ​​by different devices, the range and coefficient of variation (CV, the ratio of the standard deviation to the mean) of the measurements of each device on each indicator were calculated to eliminate the influence of the measurement scale. Second, scatter plots were generated to investigate the relationship between different measurement indicators. In addition, for the measurements of the same brand of wearable devices and mobile phone apps, correlation coefficient analysis was applied.

## Results

### Comparison of the Consistencies of the Measurements ​​of Each Indicator

**Figure 1:**
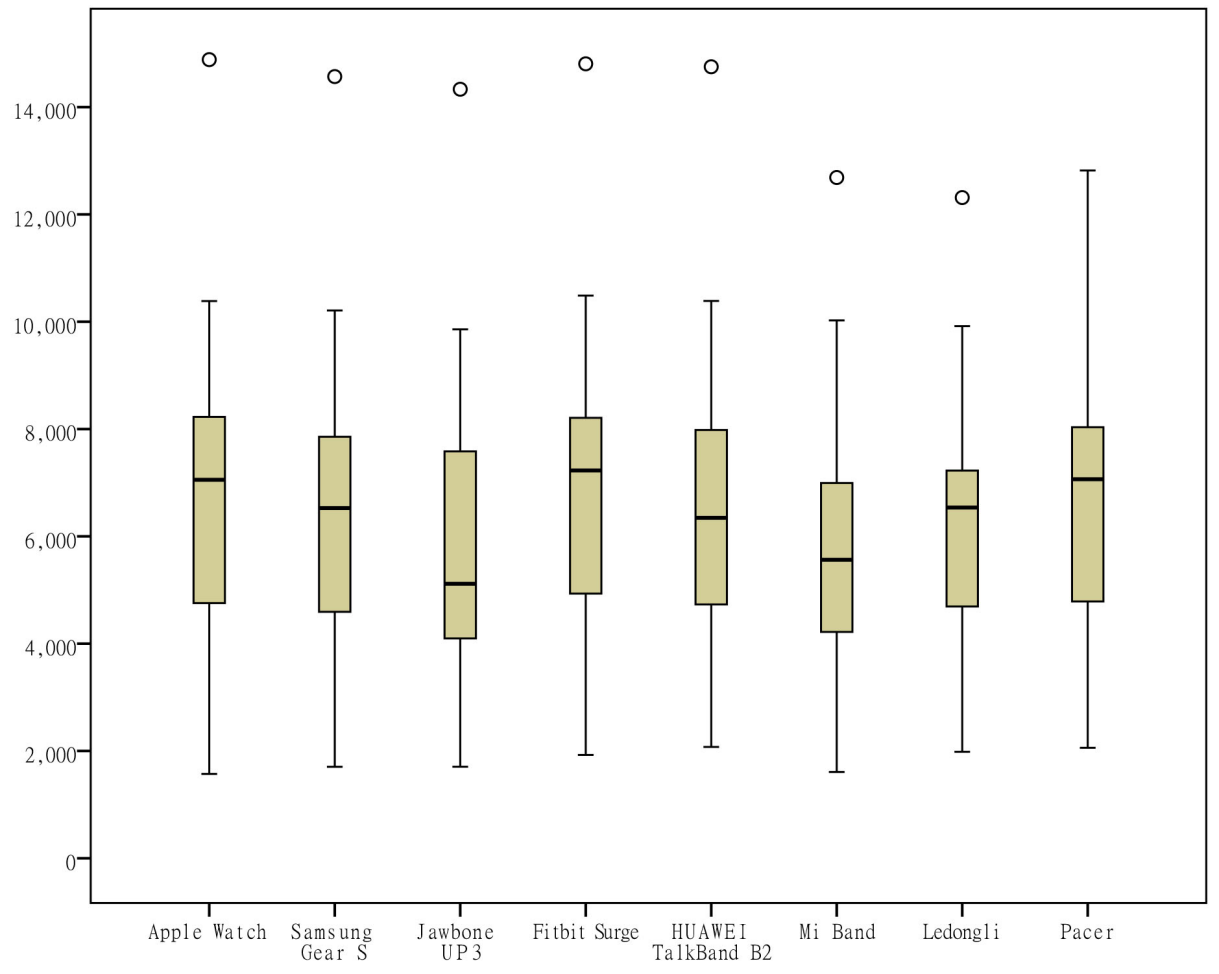
Box plot of the measurement distribution of the number of steps by different devices.

**Figure 2:**
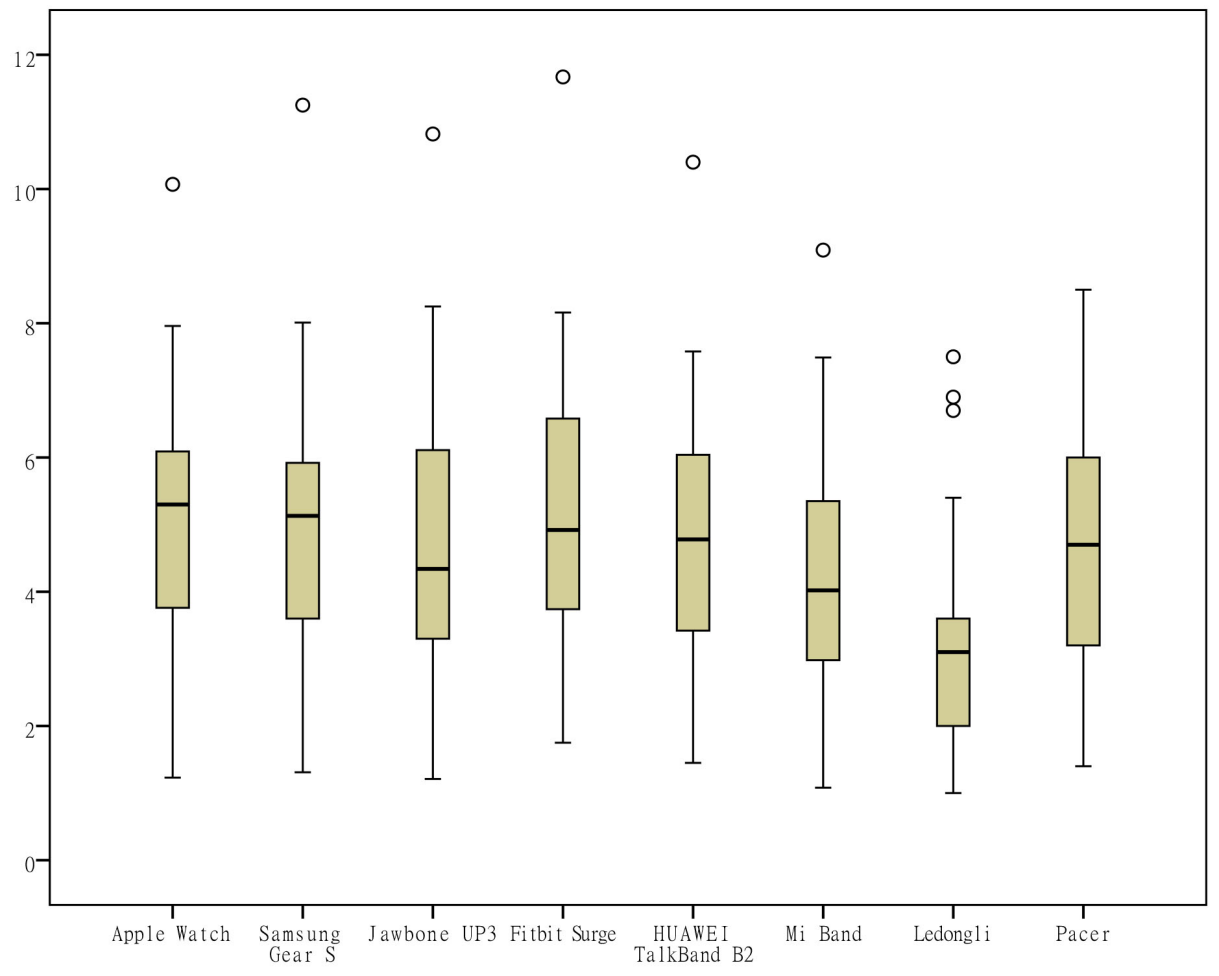
Box plot of the measurement distribution of distance by different devices.

**Figure 3:**
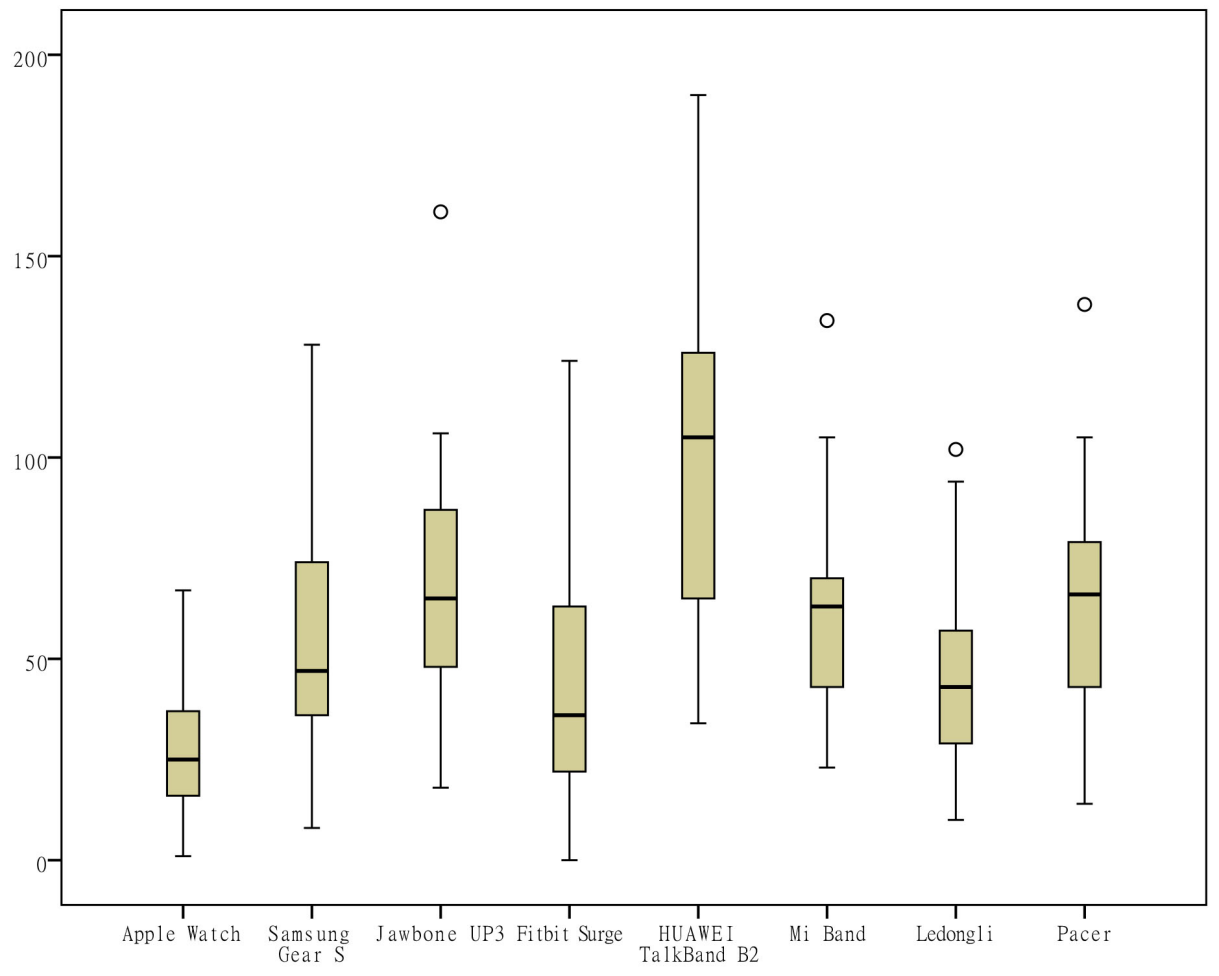
Box plot of the measurement distribution of activity duration by different devices.

**Figure 4:**
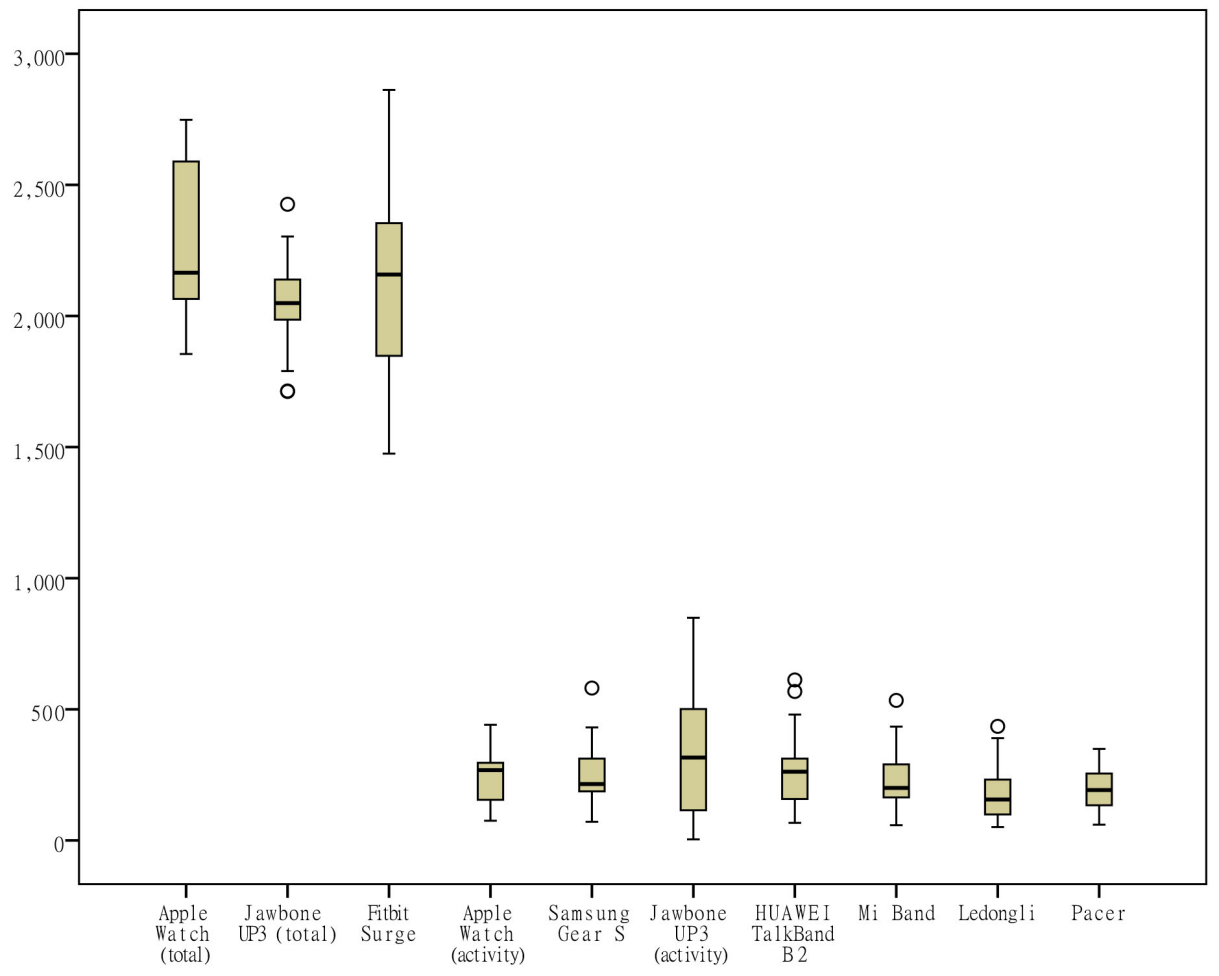
Box plot of the measurement distribution of energy expenditure by different devices.

**Figure 5:**
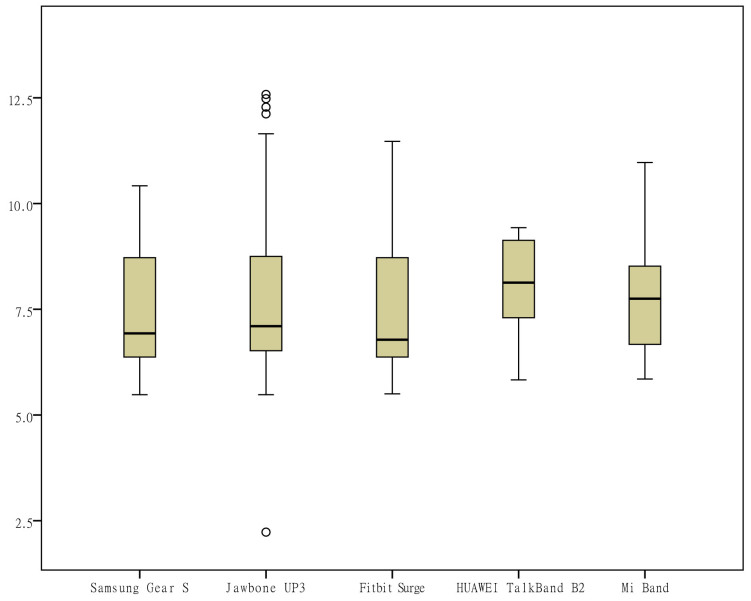
Box plot of the measurement distribution of sleep duration by different devices.

**Figure 6:**
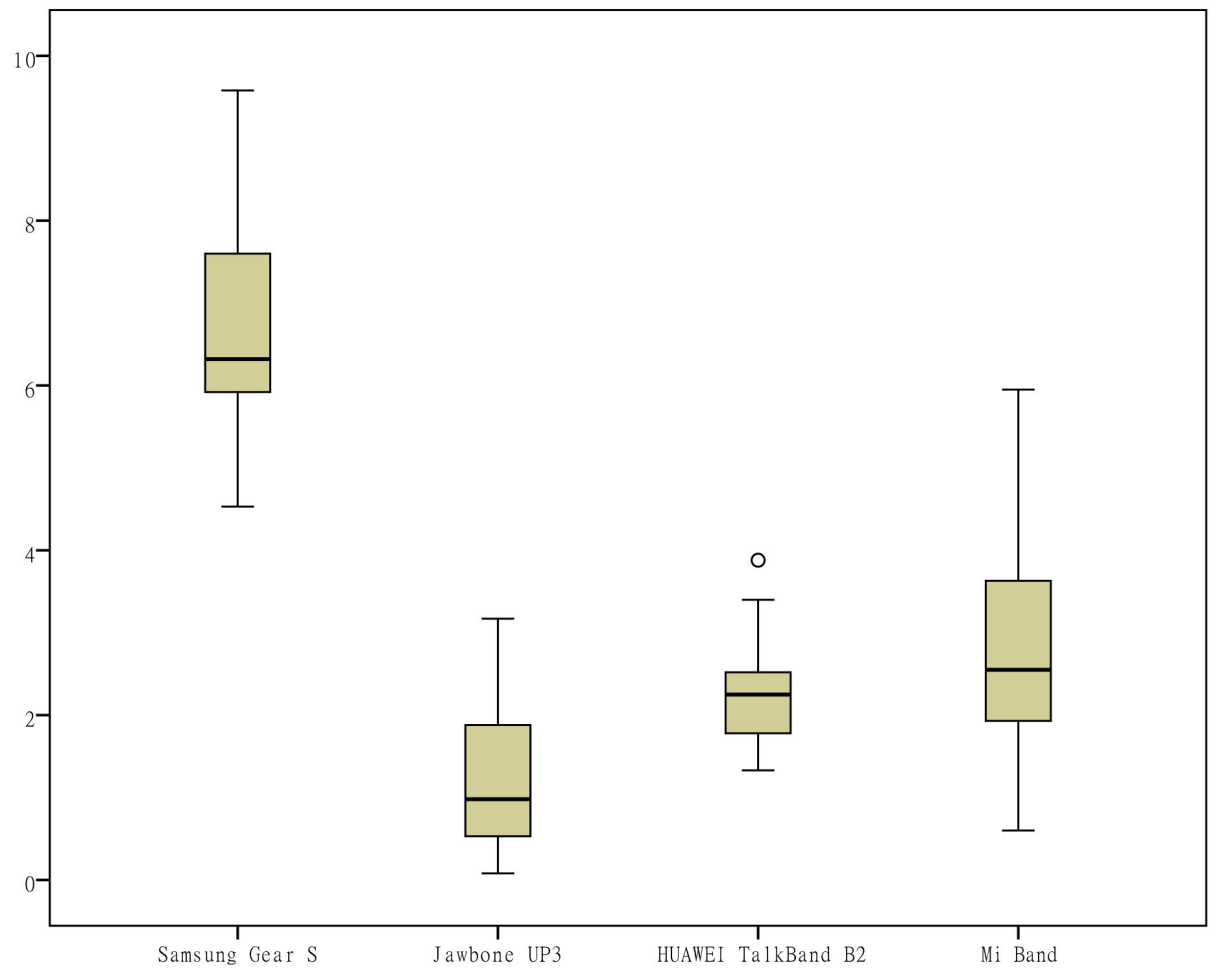
Box plot of the measurement distribution of deep sleep duration by different devices.

To visually observe the distribution differences of the measurements ​​of various indicators by the different wearable devices, box plots were generated from the measurements for the 6 indicators (the number of steps, distance, activity duration, EE, sleep duration, and deep sleep duration) according to the category of the devices (Figures 1-6). One important note to explain the outliers in the box plots is that there are 3 key points in the box plots representing the third quartile Q3, median, and the first quartile Q1, respectively. The upper limit is equal to Q3+1.5×(Q3−Q1) and lower limit is equal to Q1−1.5×(Q3−Q1). Outliers are those points that are beyond the upper or lower limits. In this case, the box-plot charts were designed to observe the overall characteristics or the consistency of the multiday measurements from different wearable devices, the outliers might be resulted from the measurement of someday when the subject was observed doing unusual activity, and therefore has no special significance.

In addition, as Apple Health and Samsung S Health apps cannot be used on the same phone at the same time, there are only 3 subjects in this study using Apple’s Health app, 2 subjects using Samsung S Health app. Therefore, taking into account different amount of data, Apple Health and Samsung S Health were not compared with other devices. The comparison of measurements by different devices from the same brand was performed later.

The differences in the measurements of the number of steps by different devices were rather small (Figure 1); the distance measurements by the app Ledongli were significantly lower, whereas those by the other devices only differed slightly (Figure 2). The activity duration measurements by different devices differed significantly (Figure 3). The EE measurements could be divided into 2 levels: the measurements by some of the devices (Apple Watch, Jawbone Up3, and Fitbit Surge) included resting EE and activity EE, whereas measurements by the other devices (Samsung Gear S, Huawei TalkBand B2, Mi Band, Ledongli, and Pacer) were specifically activity EE (Figure 4). The sleep durations and deep sleep durations were significantly different according to the different devices (Figures 5 and 6).

For each indicator, the ICC of the measurements by different wearable devices was calculated and is shown in Table 1. The ICCs of the measurements for the number of steps and distance were higher than .8, indicating excellent consistencies by different wearable devices; the ICCs of the measurements for activity duration, total EE and activity EE indicated only fair consistencies by different wearable devices; and the ICCs of the measurements for sleep duration and deep sleep duration were lower than .4, indicating poor consistencies by different wearable devices.

**Table 1:** Intraclass correlation coefficient (ICC) of the measurements by different wearable devices

Items	Intraclass correlation coefficient	95% CI
Number of steps	.89	0.83-0.94
Distance	.84	0.75-0.91
Activity duration	.59	0.44-0.75
Total EE^a^	.59	0.37-0.77
Activity EE	.57	0.41-0.74
Sleep duration	.30	0.13-0.52
Deep sleep duration	.27	0.08-0.50

^a^EE: energy expenditure.

**Figure 7:**
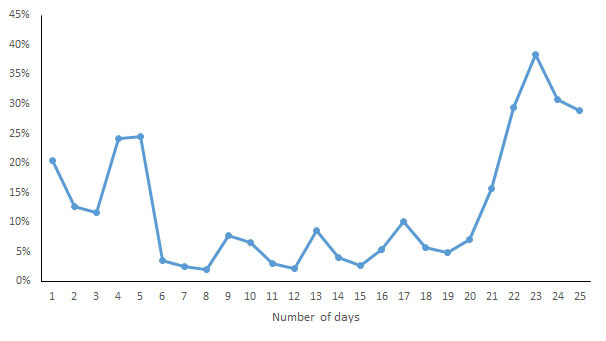
Coefficient of variation of daily measurements of the number of steps by different devices.

**Figure 8:**
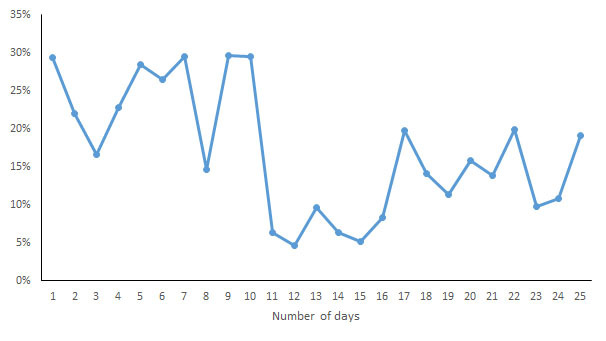
Coefficient of variation of daily measurements of distance by different devices.

**Figure 9:**
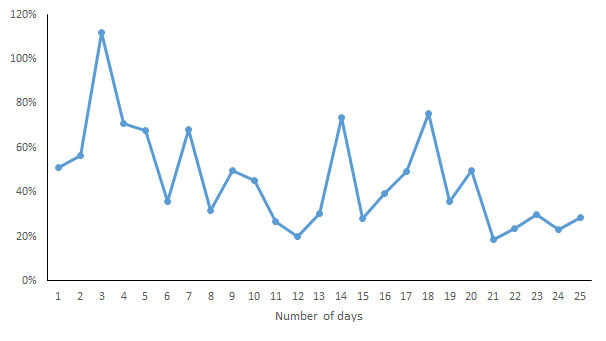
Coefficient of variation of daily measurements of active duration by different devices.

**Figure 10:**
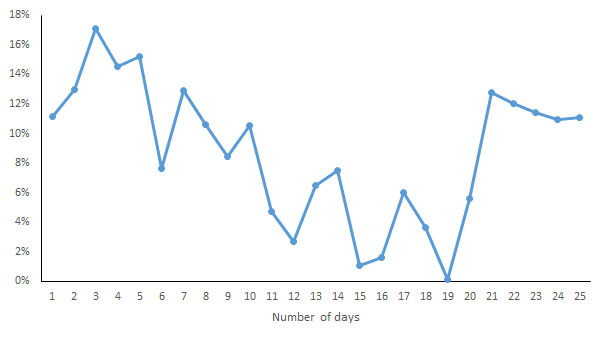
Coefficient of variation of daily measurements of total energy expenditure by different devices.

**Figure 11:**
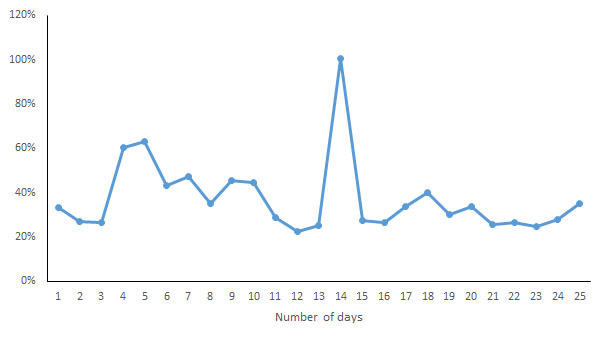
Coefficient of variation of daily measurements of activity energy expenditure by different devices.

**Figure 12:**
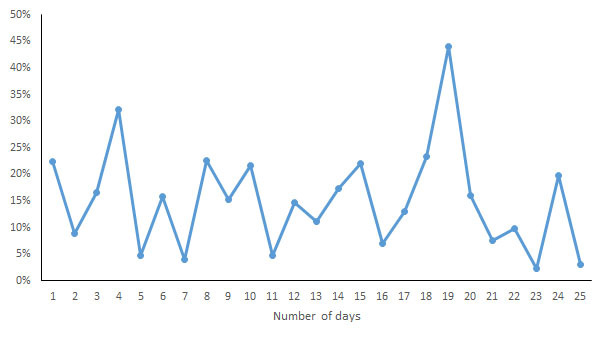
Coefficient of variation of daily measurements of sleep duration by different devices.

**Figure 13:**
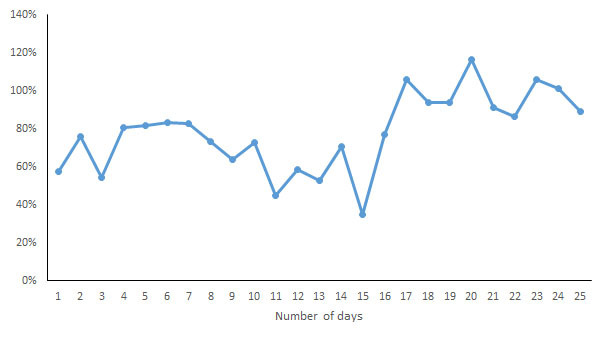
Coefficient of variation of daily measurements of deep sleep duration by different devices.

For each indicator, the CV of the daily measurements by different devices was calculated and is shown in Figures 7-13. According to the above analysis, the consistency of the number of steps was excellent; however, the CVs of the measurements of different days fluctuated greatly (2-38%), with a range of 297-8047 steps. The consistency of the distance measurements was excellent; however, the CVs of the measurements of different days also fluctuated greatly (5-30%), with the range of .4-8.7 km. For activity duration, which only had a fair consistency of measurements, the CVs of the measurements of different days varied from 19% to 112%, with a range of 22-170 min. EE also had a fair measurement consistency; the CVs of the total EE measurement of different days varied from 1% to 17%，with a range of 5-662 kcal；the CVs of activity EE measurement of different days varied from 22% to 100%，with a range of 51-706 kcal. For sleep duration and deep sleep duration, which had poor measurement consistencies, the CVs of the measurements of different days fluctuated from 2-44% and 35-117%, respectively, with a range of 29 min to 8 h 44 min for sleep duration and a range of 2 h 20 min to 7 h 40 min for deep sleep duration.

Further analysis by comparing this study to previous studies is shown in Table 2.

**Table 2:** Comparison with previous studies.

Indicators	Steps	Distance	Activity duration	EE^a^	Sleep duration	Deep sleep duration
This study	Excellent consistency with CV^b^ (2-38%)	Excellent consistency with CV (5-30%)	Fair consistency with CV (19-112%)	Fair consistency with CV for total EE (.1-17%) and activity EE CV (22-100%)	Poor consistency with CV (2-44%)	Poor consistency with CV (35-117%)
Previous studies	Consumer-grade wearable devices provided consistently similar step counts with research-grade devices for average daily activity (*P*>.05) [21]	The inter-device reliability of wearable devices in measuring distance was excellent for all treadmill speeds (ICC^c^≥.90) [14]	Consumer-level wearable devices showed moderate validity for measurement of moderate to vigorous physical activity in free-living conditions (*r*=.52-.91) [16]	Consumer-level wearable devices showed moderate validity for measurement of total daily EE in free-living conditions (*r*=.74-.81) [16]	Consumer-level wearable devices showed strong validity for measurement of sleep duration in free-living conditions (*r*>.8) [16]	Consumer-grade wearable devices showed good agreements with PSG^d^ for sleep efficiency, and they overestimated PSG sleep efficiency slightly [22]
	The inter-device reliability of wearable devices in measuring steps in free-living conditions is good (ICC≥.90) [23]	Distance errors in wearable devices were within 5% in level walking, and they overestimated distance for stair walking by at least 45% [24]	Consumer-grade wearable devices can’t accurately capture activity data across the entire 24-h day, error rates ranged from 51.8% to 92% for moderate to vigorous physical activity [17]	Consumer-grade wearable devices reasonably and reliably estimate EE during walking and running (ICC≥.95) [25]	Consumer-grade wearable devices performed consistently compared with each other (reliability=96.5-99.1%), and they overestimated sleep time by an average of 67.1 min compared with PSG [26]	Consumer-grade wearable devices performed consistently compared with each other (reliability=96.5%-99.1%), and they overestimated sleep efficiency by an average of 14.5% compared with PSG [26]

^a^EE: energy expenditure.

^b^CV: coefficient of variation.

^c^ICC: intraclass correlation coefficient.

^d^PSG: Polysomnography.

### The Relationship Between Different Measurement Indicators

**Figure 14:**
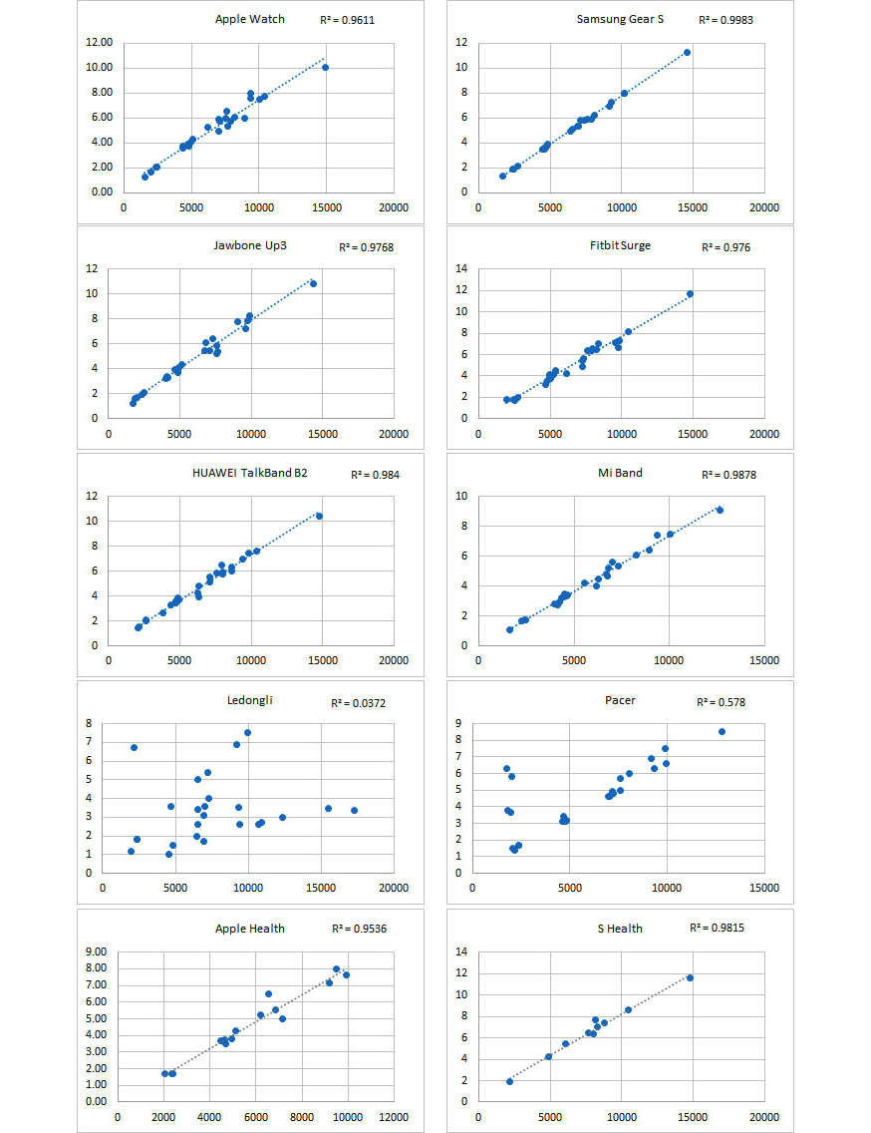
Scatter plot for each device showing measurements of the number of steps and distance.

**Figure 15:**
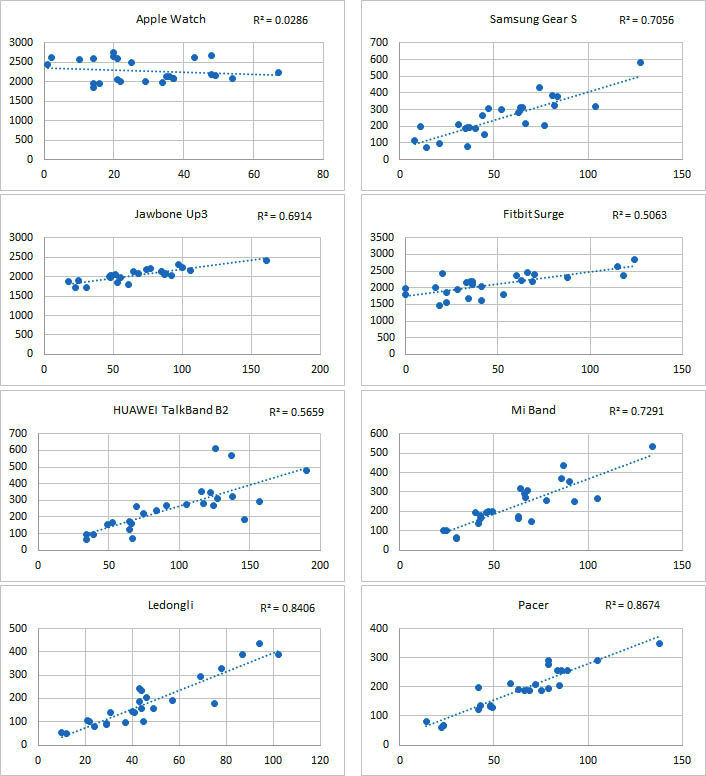
Scatter plot for each device showing the measurements of activity duration and energy expenditure.

There were correlations between the number of steps and distance and between activity duration and EE. Given that most wearable devices measure activity data based on three-axis acceleration sensors and that the ICCs of the measurements on the number of steps and distance were close, as were the ICCs of the measurements on activity duration and EE, a scatter plot was generated for each device on the number of steps and distance (Figure 14). The results showed that except for Ledongli and Pacer, the measurements of the number of steps and distance by the other devices exhibited a strong positive linear correlation (R^2^>.95), whereas the correlation between the two indicators measured by the third-party apps showed some differences. A scatter plot of activity duration and EE measurements by each device was generated and is shown in Figure 15. Except for Apple Watch, Fitbit Surge, and Huawei Wrist bracelet, the measurements of activity duration and EE by the other devices showed a positive correlation (R^2^>.7). 

### Comparison of the Consistency of Measurements ​​by Different Devices of the Same Brand

A strong correlation between different devices of the same brand was observed (r≥.88). The correlation coefficients of different measures for mobile phones and smart watches are showed in Table 3.

**Table 3:** Correlation coefficient on measurements by mobile phones and smart watches of the same brand.

Brand	Steps	Distance	EE^a^
Apple	.99	.99	.96
Samsung	.96	.91	.88

^a^EE: energy expenditure.

## Discussion

### Overview of This Study

In this study, the activity and sleep data of 5 subjects were collected on several consecutive days from the mainstream wearable devices and mobile phone apps in the state of everyday life activities to examine the consistency of the measurements by different wearable devices. The monitoring measurements by the mainstream wearable devices in the market showed a rather large daily variation on the measurements of the number of steps and distance, but the overall consistencies in the continuous measuring was excellent. Activity duration and EE displayed fair measurement consistencies, whereas the same for sleep duration and deep sleep duration were poor. In terms of the discrete level, the daily measurements on each indicator by different devices fluctuated to a certain degree. Furthermore, in all the wearable devices, there was a strong correlation between the number of steps and distance measured by the same device, and in some wearable devices and mobile apps, there was a rather strong correlation between measurements of activity duration and EE measured by the same device.

Compared with previous studies, this study has some unique innovations. First, in this study, the market representing smart watches, the internationally renowned smart bracelets, the Chinese-made smart bracelets, mobile phone health apps, and third-party health mobile phone apps were integrated in the same comparison system, which makes it possible to effectively compare the measurement differences derived from different devices under the same conditions, and one representative innovative result was that the number of steps and distance displayed excellent consistencies. The measurements by the third-party apps and the correlation between the 2 indicators were different from the values by the other devices. Second, in this study, activity and sleep data were collected from each subject for 5 days while participating in natural life activities, and all the subjects were members of the research group who were familiar with the content of the study. The continuous use of the wearable device ensured the subject to be familiar with sync rules of multiple devices, which effectively avoided the errors caused by inappropriate wearing of the device. Third, in this study, for the first time, the leading products in the market, smart watches and mobile phone apps of the same brand (Apple or Samsung), were compared in terms of measurement consistency. It is believed that products of the same manufacturer have similar technical optimization; however, it was found that the measurements by different devices of the same brand exhibited differences, likely due to factors such as hardware support and wear habits on the measurements.

However, there are 2 small limitations in this study. First, because the study was designed to monitor data in the subject’s state of normal life activities, it was difficult to obtain the gold standard through the single research-level device for each indicator. Therefore, we intended to analyze the consistency of measurements on the same indicator by different wearable devices. If the gold standard control were available, the accuracy of each device would be obtained, which will be examined in our subsequent studies. In addition, because each subject was required to continuously wear 6 pieces of wearable devices and 1 mobile phone in the daily activities of a multiday duration while observing certain rules of use, it presented a certain burden of wear for the subject; thus, only the members of the close community of our research group were recruited under the principle of convenience sampling, in a very limited sample size. A series of measures were taken to reduce selection bias and ensure a relative complete normal activity cycle of an average person, such as the emphasis of maintaining original daily activities to the participants, as well as the addition of the weekends into the study duration during which more activities might be observed. Besides, the data analysis showed that the number of steps and distance had an excellent measurement consistency; moreover, the variance homogeneities of the number of steps (*P*=.96) and distance (*P*=.85) by different wearable devices were rather good, indicating that in terms of these 2 indicators, the degrees of discrepancy of the measurements by different wearable devices were not significantly different. Therefore, the data imbalance problem arising from the sampling was not significant, and the results were valid.

### Consistency of Measurements ​​for Wearable Devices

Previous studies have shown that the measurements on the number of steps and sleep duration were reliable, whereas for other indicators, the errors were high. Dontje et al [23] asked one subject to wear 10 pieces of Fitbit Ultra in the state of natural life for 8 consecutive days and showed that when comparing minutes, hours, and days, the reliabilities of the number of steps by multiple devices were excellent and the best when compared in terms of the number of steps per day (ICC=1). Ferguson et al [16] assessed the reliabilities of the number of steps, activity duration, sleep duration, and EE of 21 subjects wearing 7 devices for 48 h in a state of natural activity and found that the measurements for the number of steps (*r*=.94-.99) and sleep duration (*r*=.82-.92) were very reliable, whereas they were fairly reliable for total EE (*r*=.74-.81) and the duration of MVPA (*r*=.52-.91); however, the reliabilities of the measurements by different devices showed significant differences. Diaz et al [25] investigated 23 subjects with 4 pieces of Fitbit One worn on the waist and 2 pieces of Fitbit Flex worn on each wrist during the activities of 4 speed phases and showed that the measurements of the number of steps by all the devices were strongly correlated with the actual number and the measurement error was underestimated by 16.3% at the highest; moreover, the measurements of EE by all the devices were strongly correlated with the actual number, and the measurement error was overestimated by 52.4% at the highest; the measurements obtained by different devices were strongly correlated and highly reliable. Bai et al [27] asked 52 subjects to wear 5 pieces of consumer-level devices and 2 pieces of research-level devices and examined the accuracy of EE measurements during optional activities of various types and found that in general, the percentage of EE measurement error ranged from 15.3% to 30.4%, and the measurements by different devices had a generally high correlation with the gold standard (.71-.90). There were 3 devices that had a percentage of EE measurement error of less than 20% when resting; 2 devices had a percentage of EE measurement error less than 20% for aerobic exercise, and for anaerobic exercise, the percentages of measurement error of all the devices were higher than 25%. Massimiliano et al [22] showed that the measurements for sleep duration, sleep efficiency, and wake-up duration by Jawbone Up exhibited excellent consistencies compared with the measurements obtained by Polysomnography (PSG), although there were still some errors and a certain degree of overestimation on sleep duration and efficiency. Meltzer et al [28] compared the accuracies of Fitbit Ultra and PSG on 63 subjects and found that in the normal mode, the sensitivity and accuracy of Fitbit Ultra were excellent but the specificity was rather poor; it significantly overestimated sleep duration and sleep efficiency. In the sensitive mode, the specificity was adequate but the sensitivity and accuracy were inadequate; it significantly underestimated sleep duration and sleep efficiency. The above studies show that the reliability of the measurement on the number of steps was rather high, whereas the measurements for activity duration and EE had a high error; the measurements for sleep were rather reliable, although in general, sleep duration was overestimated.

The conclusions of this study are consistent with the conclusions of previous studies but differ regarding sleep duration. This study found that the measurements for the number of steps and distance were reliable, whereas the measurements for the other indicators were not; however, previous studies found that the measurements on sleep duration were reliable. Further analysis of the CVs of the daily measurements on sleep duration by each device revealed that the average CV for all days was 17% and that of deep sleep duration, which also had a poor measurement consistency, was 74%. The data showed that the measurements on sleep duration by different devices varied little, leading to a low CV, which is consistent with the findings in previous reports that the errors in the measurement of sleep duration by different devices were low. However, the relative values of the measurements by different devices on different times fluctuated, leading to unstable performances of different devices in repeated measurements and poor measurement consistencies.

### Significance of Measurements by Wearable Device

The number of steps and distance are reliable indicators of health evaluation for wearable devices. Studies have previously shown that daily activities such as walking and sitting showed a significant causal relationship with health and chronic condition rehabilitation [3-5]. In this study, we found that except for Ledongli, the consistencies of the measurements of number of steps and distance were excellent and could provide reliable judgment on the individuals’ activity amount. Furthermore, except for Ledongli and Pacer, the measurements of the number of steps and distance showed a strong correlation, likely because distance was calculated from a linear function based on the total number of steps. However, in each device, there were discrete values that deviated from the regression line that were mainly derived from differences in the subjects’ activity habits. The activity monitoring sensor of the devices included in this study was mainly the three-axis accelerometer, and it is believed that in addition to the users’ initial base data that must be collected, the dynamic data on which the monitoring rely were generated from the real-time signal change of the acceleration. Moreover, the fitting function of each device was different, and the measurements by different devices differed due to factors such as the included variables, algorithm models, and so on.

Wearable devices are less reliable for measuring activity duration and EE. A variety of wearable devices do not specify activity duration. Academic studies often determine the presence of MVPA based on the metabolic equivalent of energy (EE at rest or sitting) [27,28]. Thus, moderate physical activity refers to 3-6 METs, requiring a moderate degree of motion and significant heart rate acceleration; vigorous physical activity refers to more than 6 METs, requiring a large amount of movement that lead to rapid breathing and a rapid increase in heart rate [29]. Due to their health benefits, MVPA are often used as a public health indicator to assess the level of activity in the population [27,28]. This study also found that activity duration and EE exhibited a strong correlation in the majority of devices, but the consistencies of the measurements on activity duration and EE by each device were rather poor, indicating that the 2 indicators are not suitable for the evaluation of activity. In addition, some of the devices differentiated resting EE and activity EE, and most of the devices only measured activity EE, whereas resting EE could actually reach 2000-3000 kcal, and the EE of everyday activity was less than 500 kcal. Basal metabolism may be affected by diet, temperature, endocrine factors, and so on; thus, resting EE is a dynamic value under the action of a variety of internal and external environmental factors. However, wearable devices mainly monitor the state of motion, so it is still doubtful whether the current wearable devices are able to provide information on resting EE.

Wearable devices are rather rudimentary on monitoring sleep. According to the Rechtschaffen and Kales classification, sleep can be divided into the rapid eye movement phase and the nonrapid eye movement phase; the fourth period of the nonrapid eye movement phase is the deep sleep stage, which has the high amplitude brain wave, mainly the delta-wave with a frequency of 1-2 times/s, and promotes physical and mental recovery [30]. The majority of the devices in this study could automatically determine the time points at which the user fell asleep and woke up; however, the measured sleep duration increased at varying degrees compared with the standard reference sleep duration, likely because of the level of activity on the bed during the time periods of falling asleep and waking up, which led to the device misreading the time periods as still being asleep. A few wearable devices were rather reliable in determining sleep duration, but the consistencies of the measurements by various devices were inadequate, and this function requires further calibration.

### Conclusions

The consistencies of the number of steps and distance by wearable devices were excellent, and the 2 indicators can be used in health evaluations, whereas the consistencies of the measurements on activity duration, EE, sleep duration, and deep sleep duration were only fair or poor. These will directly affect consumers’ acceptance of wearable devices and require the manufacturers’ close attention and resolution as well.

## Abbreviations

BMI: body mass index
CV: coefficient of variation
EE: energy expenditure
ICC: intraclass correlation coefficient
MET: metabolic equivalent of energy
MVPA: moderate to vigorous physical activity
PSG: polysomnography
